# Gray Matter Asymmetry Alterations in Patients With Spinocerebellar Ataxia Type 3: A Voxel‐Based Morphometric Comparison Study

**DOI:** 10.1111/cns.70171

**Published:** 2024-12-25

**Authors:** Linfeng Shi, Lan Ou, Peiling Ou, Lihua Deng, Yonghua Huang, Xingang Wang, Li Gui, Bijia Wang, Limeng Dai, Guolin Ma, Jian Wang, Chen Liu

**Affiliations:** ^1^ 7T Magnetic Resonance Imaging Translational Medical Center, Department of Radiology, Southwest Hospital Army Medical University (Third Military Medical University) Chongqing China; ^2^ Department of Neurology, Southwest Hospital Army Medical University (Third Military Medical University) Chongqing China; ^3^ Prenatal Diagnosis Center, Department of Gynecology and Obstetrics, Southwest Hospital Third Military Medical University (Army Medical University) Chongqing China; ^4^ Department of Radiology China‐Japan Friendship Hospital Beijing China

**Keywords:** brain, brain asymmetry, gray matter, magnetic resonance imaging, spinocerebellar ataxia type 3

## Abstract

**Aims:**

The aim of this study was to investigate the whole‐brain asymmetry changes in spinocerebellar ataxia type 3 (SCA3) and their association with movement disorders.

**Methods:**

Voxel‐based morphometry (VBM) was used to assess asymmetry in gray matter (GM) volume in 83 genetically confirmed SCA3 patients and 83 sex‐ and age‐matched healthy controls (HCs). The asymmetry index (AI) was analyzed for partial correlation with disease severity, as measured by the Scale for Assessment and Rating of Ataxia (SARA) and International Cooperative Ataxia Rating Scale (ICARS). Age, sex, and total intracranial volume (TIV) were included as covariates in the analysis.

**Results:**

Asymmetry in GM analysis with SCA3 patients showed decreased leftward asymmetry in cerebellar lobules VIII and IX, the visual cortex, and the putamen, as well as decreased rightward asymmetry in the ventral lateral thalamus, as analyzed by VBM. The AI in the cerebellum, the visual cortex, and the putamen was positively correlated with SARA and ICARS scores, whereas the AI in the thalamus was negatively correlated with these scales.

**Conclusion:**

SCA3 patients exhibit distinct patterns of asymmetrical changes in GM volume, which correlates with motor dysfunction. These changes in asymmetry may serve as potential biomarkers for early intervention in SCA3.

**Trial Registration:**

Chinese Clinical Trial Registry (ChiCTR): 1800019901, 2000039434

## Graphical abstract

Patients with SCA3 exhibit asymmetric cerebellar atrophy, particularly more pronounced on the left side, which is associated with motor balance impairments. Additionally, bilateral asymmetric atrophy is observed in the brain, correlating with motor dysfunction.

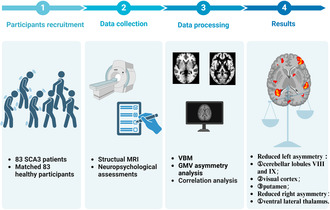

## 1 Introduction

Structural asymmetry is a fundamental aspect of human brain organization, underlying various functions. Differences between the left and right hemispheres are evident in several domains, including cognitive function, motor control, emotional regulation, and visuospatial attention [1, 2, 3, 4]. This asymmetry is considered an evolutionary advantage, enhancing the efficiency of neural processing. The typical pattern of asymmetry emerges during fetal development and is associated with variations in gene expression and protein distribution between the hemispheres [2, 5]. Multiple factors, including development, environment, sex, and genetics, influence this asymmetry [6, 7]. Alterations in typical brain asymmetry have been reported in various brain disorders [8], such as schizophrenia [9, 10], autism spectrum disorder (ASD) [11, 12], Parkinson's disease (PD) [3], Friedreich's ataxia (FRDA) [13], Huntington's disease (HD) [14], and stroke [15]. Research indicates that gray matter (GM) asymmetry caused by these diseases is not confined to the cerebrum; the cerebellum also exhibits changes. Schizophrenia patients show a more pronounced asymmetry in the volume of cerebellar hemispheres [9]. Gray asymmetries in the cerebellum and associated cortical areas are linked to motor coordination difficulties and cognitive deficits in disorders like ADHD [4]. In stroke patients, balance functions dominated by the cerebellum negatively correlate with indices of brain asymmetry. However, the effects of cerebellar diseases, such as spinocerebellar taxia type 3 (SCA3), on overall brain GM asymmetry remain poorly understood.

SCA3 is a neurodegenerative disorder primarily characterized by cerebellar atrophy. Clinically, it presents as progressive cerebellar ataxia along with motor and nonmotor symptoms, such as impairment of gait, extremity coordination, disordered eye movements, and impairments of intellectual function and emotional or psychiatric disturbances [16, 17]. SCA3 is caused by an abnormal expansion of CAG repeats in exon 10 of the *ATXN3* gene on chromosome “14q32.1,” leading to the elongation of polyglutamine chains and the abnormal deposition of ATXN3 protein [18, 19]. Previous studies have revealed widespread structural abnormalities in the brains of SCA3 patients [20, 21, 22]. However, it remains unclear whether bilateral hemispheres are equally affected and whether this inconsistency is associated with the disease process.

We hypothesized that GM volume asymmetry is altered in patients with SCA3, and that the severity of motor and nonmotor symptoms is associated with this alteration. To test this hypothesis, we employed voxel‐based morphometry (VBM) [23], which has been widely and successfully used to explore asymmetric changes in brain structure across various neurological and psychiatric disorders [24, 25, 26]. The aim of this study was to identify unique changes in brain structural asymmetry that could serve as a biomarker to assess disease progression and explore the pattern of asymmetric changes throughout the brain in cerebellar atrophy disease.

## 2 Materials and Methods

### 2.1 Subjects

The research was approved by the Medical Ethics Committee of the First Affiliated Hospital of Army Medical University (Nos: KY2020191, KY2023046). Written informed consent was obtained from all participants prior to their inclusion in the study. This study was based on the research registered with the Chinese Clinical Trial Registry. Participants were recruited from the First Affiliated Hospital of Army Medical University between 2018 and 2023. Participants were enrolled if they (1) aged > 18 years and (2) were genetically diagnosed with SCA3 (for patients). Exclusion criteria for SCA3 patients included (1) history of psychiatric disorders, traumatic brain injury, metabolic disorders, chronic medical conditions (e.g., heart failure), or other neurological diseases besides SCA3; (2) contraindications for MR examination (e.g., claustrophobia, ferromagnetic metal implants); (3) left‐handed individuals—as they might introduce biases in brain lateralization analysis; (4) incomplete MRI data or clinical assessment; and (5) poor image quality (e.g., significant motion artifacts). Exclusion criteria for the normal control group included (1) any of the above exclusion criteria and (2) cognitive impairment (Montreal Cognitive Assessment [MoCA] < 26). This study followed the Strengthening the Reporting of Observational Studies in Epidemiology (STROBE) reporting guideline (eMethods in the Supporting Information).

### 2.2 Neuropsychological Assessments

Neuropsychological assessments were independently evaluated by the two neurologists, including the Scale for the Assessment and Rating of Ataxia (SARA), the International Cooperative Ataxia Rating Scale (ICARS), the MoCA, Activities of Daily Living (ADL), Instrumental Activities of Daily Living (IADL), Rapid Verbal Retrieval (RVR), the Digit Span Test (DST), and the Hamilton Depression Scale (HAMD).

### 2.3 MRI Acquisition

A Siemens 3.0‐Tesla Magnetom Trio MRI scanner (Siemens Healthcare, Erlangen, Germany) was employed for whole‐brain imaging using a 12‐channel head coil. Participants were scanned in a supine position with their heads secured by foam pads to reduce movement, and they were instructed to remain as still as possible during the procedure. Structural imaging was performed with a three‐dimensional magnetization‐prepared rapid gradient echo (3D‐MPRAGE) sequence, with the following settings: repetition time (TR) = 1900 ms, echo time (TE) = 2.52 ms, inversion time (TI) = 900 ms, flip angle = 9°, field of view (FOV) = 256 × 256 mm, slice thickness = 1.0 mm, number of slices = 176, and voxel dimensions = 1 mm × 1 mm × 1 mm.

### 2.4 MRI Analysis

Structural image preprocessing was conducted using Statistical Parametric Mapping 8 (SPM8; https://www.fil.ion.ucl.ac.uk/spm/software/spm8/) and Voxel‐based Morphometry 8 (VBM8; http://dbm.neuro.uni‐jena.de/vbm8) toolkit on a MATLAB platform (R2022a). GM asymmetry was assessed through a predefined voxel‐based asymmetry analysis protocol, which involved several critical steps [27]. Initially, all T1‐weighted images underwent visual inspection to confirm their suitability for subsequent analysis. The T1 images were then segmented into gray and white matter compartments and registered to MNI space using a 12‐parameter affine transformation. The resulting affine‐registered gray and white matter images were flipped along the sagittal midline. These original and flipped affine‐registered images were then used to generate a symmetric Diffeomorphic Anatomical Registration Through Exponentiated Lie Algebra (DARTEL) template. The GM images, both flipped and original, were registered to the symmetric DARTEL template.

### 2.5 Asymmetry Index Calculation

The asymmetry index (AI) on each voxel was calculated using the following formula: AI = ([Right−Left]/[0.5 × (Right + Left)]) [27]. The left hemisphere images were discarded, leaving only the right hemisphere, which was smoothed with an 8 mm full‐width at half‐maximum Gaussian kernel. The resulting smoothed right hemisphere AI images were used for subsequent statistical analysis. The positive AI values in the right hemisphere indicate rightward asymmetry, whereas the negative AI values indicate leftward asymmetry.

### 2.6 Statistical Analysis

The demographic and clinical features of patients with SCA3 and HCs were compared using SPSS 22.0. The Shapiro–Wilk test was applied to assess the normality of the data distributions. Data with a normal distribution were expressed as mean ± SD ($\bar{x}\pm s$) and analyzed using independent samples *t* tests. Data not conforming to a normal distribution were reported as median (*M*) with interquartile range (*Q1*, *Q3*) and analyzed using the Mann–Whitney *U* test. Gender distribution differences were evaluated with the chi‐square test. All tests of significance were two‐tailed, and *p* value < 0.05 was considered to be statistically significant.

The statistical analyses of AI were conducted using the SPM software package. The voxel‐wise GM asymmetry differences between SCA3 and HCs groups were examined via a general linear model with the covariates of interest, including age, sex, and total intracranial volume (TIV). To correct for multiple comparisons, we applied a cluster‐based Familywise Error Correction (FWE) approach. Specifically, an initial voxel‐wise threshold of *p* < 0.001 (*T* > 3.14) was used to define clusters of contiguous voxels. A minimum cluster size of 40 was applied to ensure robust cluster identification. Next, clusters were evaluated for significance at the cluster level, with a cluster‐defining threshold of *p* < 0.05. For the subsequent analysis, the AI and bilateral average GM volume in the regions exhibiting significant differences were extracted. Partial correlation analysis was conducted to explore associations between the AI values extracted from the significantly different GM regions and clinical scales, controlling for age, sex, and TIV (*p* < 0.05, two‐tailed).

## 3 Results

### 3.1 Participants' Characteristics

**FIGURE 1:**
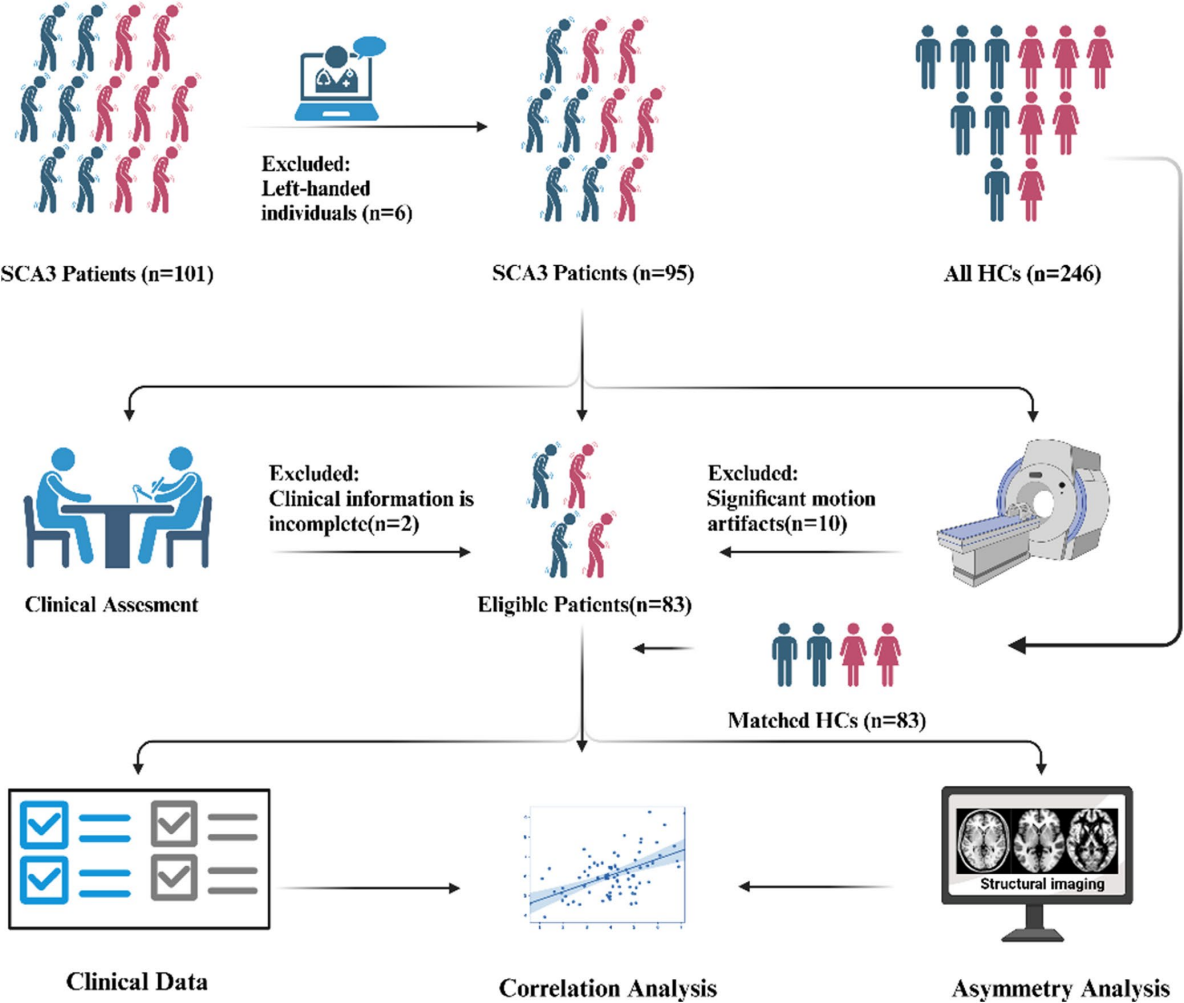
This figure provides a visual summary of the study's workflow, detailing each step from sample collection to data analysis.

The study analysis included 83 participants with SCA3 (mean age 42.27 ± 12.21 years, 46 males) after the exclusion of 18 of the 101 patients. Exclusions were due to left‐handed individuals (*n* = 6), clinical information is incomplete (*n* = 2) and significant motion artifacts (*n* = 10), as detailed in Figure 1. The study also included 83 healthy controls (HCs) matched for sex and age (mean age 42.35 ± 12.76 years, 48 males), selected using the Propensity Score Matching (PSM) method from a pre‐existing database of 246 HCs. The demographic information and clinical data for the SCA3 and HCs groups are summarized in Table 1. No statistical differences were found in age and gender between the two groups (both *p* > 0.05). Patients with SCA3 had lower MoCA scores than the HCs (*p* < 0.001).

**TABLE 1:** Demographic and clinical characteristics of patients with SCA3 and HCs.

	SCA3 (*n* = 83)	HCs (*n* = 83)	*T*/*Z*/*X* ^2^	*p*
Sex (male/female)	46/37	48/35	0.098	0.754^a^
Age (years)	42.27 ± 12.21	42.35 ± 12.76	0.044	0.965^b^
MoCA score	24.00 (20.00–28.00)	28.00 (27.00–29.00)	−6.047	< 0.001*** ^,^ ^c^
Disease duration (years)	6.00 (2.00–10.00)	—	—	—
CAG repeats	68.00 (65.00–74.00)	—	—	—
SARA score	10.00 (6.00–13.00)	—	—	—
ICARs score	27.00 (16.00–35.00)	—	—	—
ADL + IADL score	22.00 (20.00–34.00)	—	—	—
RVR score	40.00 (30.00–48.00)	—	—	—
DST score	8.00 (7.00–10.00)	—	—	—
HAMD score	6.00 (1.00–9.00)	—	—	—

*Note:* Values are presented as ranges (mean ± SD or median with interquartile range).

Abbreviations: ADL, activities of daily living; disease duration, time between onset and examination; DST, digit span test; HAMD, Hamilton depression rating scale; HCs, health controls; IADL, instrumental activities of daily living; ICARs, International Cooperative Ataxia Rating Scale; MoCA, montreal cognitive assessment; RVR, rapid verbal retrieval; SARA, scale for the assessment and rating of ataxia; SCA3, spinocerebellar ataxia type 3.

^a^ Two‐tailed Pearson chi‐square test.

^b^ Two‐sample two‐tailed *t* test.

^c^ Mann–Whitney *U* test.

*** *p* < 0.001.

### 3.2 Asymmetry

**FIGURE 2:**
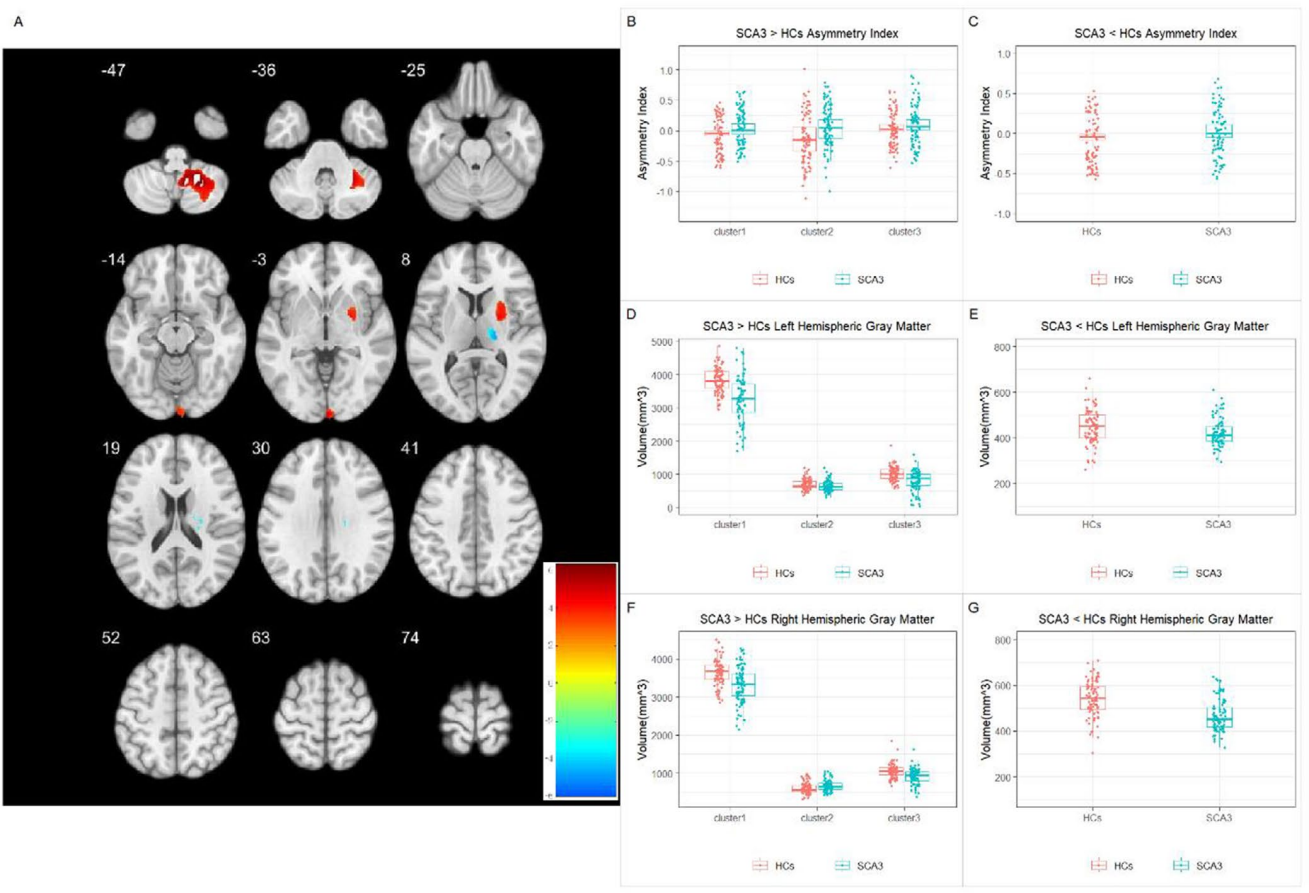
Changes in gray matter asymmetry in patients with SCA3. (A) Regions with altered gray matter asymmetry in SCA3 patients compared to healthy controls; red indicates increased rightward asymmetry, whereas blue indicates decreased rightward asymmetry. The regions showing significant gray matter volume asymmetry changes include the cluster 1 (cerebellar lobules VIII and IX), cluster 2 (visual cortex), cluster 3 (putamen), and cluster 4 (ventral posterolateral thalamus).(B, C) Comparisons of asymmetry index (AI) in regions with significant differences. Positive AI values indicate rightward asymmetry, whereas negative AI values indicate leftward asymmetry.(D, E) Comparisons of mean gray matter volume in the left hemisphere in regions with significant differences. (F–G) Comparisons of mean gray matter volume in the right hemisphere in regions with significant differences.

The VBM group analysis revealed left‐biased lateralization of GM loss in the cerebellar lobules VIII and IX, visual cortex, and putamen. However, there was a right‐biased lateralization of GM loss in the ventral posterolateral thalamus. Comparison of average volumes in significantly different regions revealed that the SCA3 patient group had decreased bilateral GM volumes in the cerebellum and putamen relative to the HCs, with a more pronounced reduction on the left side. In the visual cortex, the right side showed increased GM volume compared to the HCs, whereas the left side exhibited a decrease. For the thalamic region, bilateral GM volumes were reduced compared to the HC group, with a more significant reduction on the right side (Figure 2 and Tables 2 and 3).

**TABLE 2:** Group differences in gray matter asymmetry.

Cluster	*p*	*T* statistic	Cluster size	MNI coordinates (*X*, *Y*, *Z*)	Brain region
SCA3 > HCs					
1	< 0.001	6.277	2082	36, −58, −33	Cerebellar tonsil, cerebellar lobules VIII and IX
2	0.030	4.590	398	4, −100, 4	Visual cortex
3	0.002	4.441	724	24, 12, 4	Putamen
SCA3 < HCs					
4	0.021	−5.941	436	24, −16, 24	Ventral posterolateral thalamus

*Note:* Results are reported using a voxel‐wise threshold of *p* < 0.001 and a cluster‐size FWE threshold of *p* < 0.05.

Abbreviations: HCs, healthy controls; SCA3, spinocerebellar ataxia type 3.

**TABLE 3:** Group comparison of intrahemispheric GM volumes in regions with significant differences.

Cluster	*p*	
	Left hemisphere	Right hemisphere
SCA3 > HCs		
1	< 0.001***	< 0.001***
2	0.043*	0.002**
3	< 0.001***	< 0.001***
SCA3 < HCs		
4	0.007**	< 0.001***

*Note:* ANCOVA results comparing gray matter volumes in significant clusters between SCA3 and HCs, using total intracranial volume as a covariate for intrahemispheric differences. cluster 1: cerebellum; cluster 2: visual cortex; cluster 3: putamen; cluster 4: ventral posterolateral thalamus.

Abbreviations: HCs, healthy control; SCA3, spinocerebellar ataxia type 3.

* *p* < 0.05.

** *p* < 0.01.

*** *p* < 0.001.

### 3.3 Correlation Analysis

**FIGURE 3:**
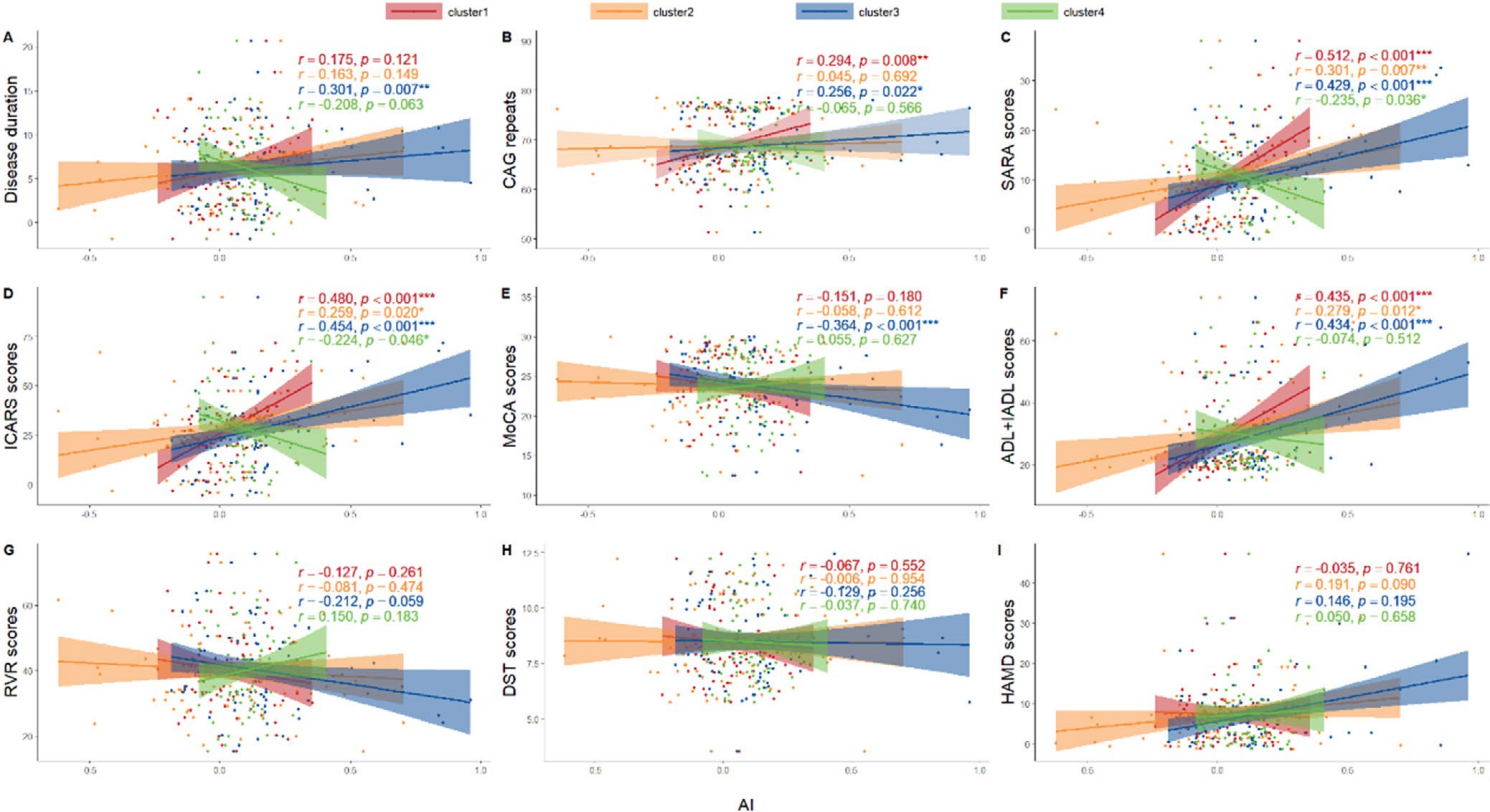
Correlation of asymmetry index with motor and neuropsychological features in a SCA3 patient. The results are adjusted for age, sex, and total intracranial volume. The x‐axes represent the asymmetry index. The regions showing significant gray matter volume asymmetry changes include cluster 1 (cerebellar lobules VIII and IX), cluster 2 (visual cortex), cluster 3 (putamen), and cluster 4 (ventral posterolateral thalamus). ADL, activities of daily living; DST, digit span test; HAMD, Hamilton depression scale; IADL, instrumental activities of daily living; ICARs, International Cooperative Ataxia Rating Scale; MoCA, montreal cognitive assessment; RVR, rapid verbal retrieval; SARA, scale for the assessment and rating of ataxia;*p < 0.05, **p < 0.01, ***p < 0.001.

After controlling for the confounders of sex, age, and TIV, the partial correlation analysis showed that the mean AI of the cerebellum was positively correlated with CAG repeat length (*p* = 0.008, *r* = 0.294), SARA total score (*p* < 0.001, *r* = 0.512), ICARS total score (*p* < 0.001, *r* = 0.480), and ADL + IADL scores (*p* < 0.001, *r* = 0.435). Similarly, the mean AI of the visual cortex was positively correlated with SARA total score (*p* = 0.007, *r* = 0.301), ICARS total score (*p* = 0.020, *r* = 0.259), and ADL + IADL scores (*p* = 0.012, *r* = 0.279). The mean AI of the putamen showed positive correlations with disease duration (*p* = 0.007, *r* = 0.301), CAG repeat length (*p* = 0.022, *r* = 0.256), SARA total score (*p* < 0.001, *r* = 0.429), ICARS total score (*p* < 0.001, *r* = 0.454), and ADL + IADL scores (*p* < 0.001, *r* = 0.434), whereas it was negatively correlated with MoCA scores (*p* = 0.001, *r* = −0.364) (Figure 3). The mean AI of the thalamus was negatively correlated only with SARA total score (*p* = 0.036, *r* = −0.235) and ICARS total score (*p* = 0.046, *r* = −0.224) (Figure 3).

## 4 Discussion

This study focuses on the asymmetry of GM volume in patients with SCA3 and reveals significant alterations in the degree of asymmetry across multiple brain regions. The cerebellum is the most significantly affected region, with varying degrees of involvement observed in the cerebral cortex and subcortical structures. Our findings confirm that these changes are closely tied to disease progression. The worsening of motor balance dysfunction is associated with asymmetric cerebellar atrophy, whereas bidirectional brain asymmetric atrophy also plays a role in the disease progression.

We have discovered left‐biased lateralization of GM loss in cerebellar lobules VIII and IX, the most affected region in SCA3 patients. This result indicates that these motor‐related regions of the cerebellum undergo the most pronounced degeneration during disease progression [28, 29]. Healthy individuals also exhibit both functional and structural asymmetries in the cerebellum. Typically, the right cerebellar hemisphere is functionally specialized for visuospatial tasks, whereas the left hemisphere is more involved in language functions [30, 31]. Specifically, the part of the anterior lobe, along with lobules VIIIA and IX, displays significantly greater volumes on the left side [30]. Our results confirm that the usual leftward GM asymmetries in cerebellar lobules VIII and IX are diminished in SCA3 patients, potentially leading to more pronounced lateralized dysfunction. Furthermore, a strong correlation was observed between the altered cerebellar lateralization and motor function decline. Asymmetric cerebellar damage is closely linked to motor dysfunction in various brain disorders, as demonstrated in previous studies [32]. In patients with PD, lateralized atrophy of the cerebellar lobules VIII–X is linked to declines in cognitive function and motor instability [33]. Similarly, a research on HD has shown asymmetrical cerebellar atrophy, with particularly severe left‐sided atrophy of the VIIb and VIIIa lobules, which is associated with reduced cerebellar function specificity [34]. Additionally, in patients with ALS, asymmetric GM volume atrophy was observed in the cerebellar posterior lobes and vermis, particularly on the left side, and this was closely associated with motor function impairments [35]. Our findings further confirm that SCA3 exhibits a left‐sided lateralized damage pattern in the cerebellum similar to that observed in other diseases.

The bidirectional pattern of brain asymmetry, particularly involving the basal ganglia and thalamus, is a defining feature of brain abnormalities in SCA3 patients. This indicates that the degenerative process in SCA3 extends beyond the cerebellum, affecting the broader cortical‐basal ganglia‐cerebellar‐thalamic‐cortical (CSTC) circuit [36, 37]. This circuit plays a critical role in motor control, and its integrity may be compromised as these regions atrophy, potentially leading to significant motor dysfunction [38, 39]. Our previous study demonstrated that white matter damage exacerbates motor control imbalance in SCA3 patients. Specifically, the structural connectivity of the cerebellar‐thalamo‐cortical (CTC) tract is significantly reduced, whereas the connection of the cortico‐ponto‐cerebellar (CPC) tract is noticeably strengthened [40]. Additionally, asymmetry in the CSTC circuit may be closely related to the severity of motor symptoms in various neurological disorders. For instance, asymmetric abnormalities in dopaminergic function within the putamen are considered a potential factor contributing to motor impairments in patients with PD [33]. Similarly, a study about HD showed that degenerative changes in the putamen and reduced left‐sided asymmetry of its GM volume are closely related to disease progression [14]. We also observed that the reduction in asymmetry of the thalamus and putamen in SCA3 patients correlates with the severity of motor symptoms, indicating that the dysfunction of these associated neural networks may exacerbate motor deficit. Our study identifies a distinctive pattern of right‐lateralized thalamic degeneration in SCA3, differing from the leftward asymmetry commonly observed in other neurodegenerative diseases [41, 42]. This rightward asymmetry might result from disruptions in the CTC pathway [43], which connects the cerebellum—especially the neocerebellum—to the contralateral cerebral cortex. Within this pathway, cerebellar output primarily reaches the contralateral ventrolateral thalamic nucleus, which then projects to the motor cortex to support fine motor coordination and motor planning [38, 44]. In SCA3, cerebellar degeneration may alter this pathway, resulting in structural and functional changes specifically within the contralateral ventrolateral nucleus, consistent with the asymmetric degeneration pattern observed in our findings.

Our study found increased GM volume in the right visual cortex and decreased volume in the left, leading to reduced left‐lateralized asymmetry. This contrasts with other brain regions. This altered asymmetry may reflect a compensatory response to cerebellar dysfunction, as the brain reconfigures visual‐motor processing networks to maintain function despite degeneration. Such reorganization has been observed in various neurodegenerative diseases [45]. Additionally, our previous DTI study indicated that enhanced asymmetric cortical‐cerebellar connectivity, a form of compensatory change, was negatively correlated with motor dysfunction in SCA3 patients [40]. Changes in the cerebellar‐visual cortex network in functional studies may further enhance visual–spatial integration [46]. These findings suggest that compensatory mechanisms may contribute to the observed asymmetry patterns in SCA3.

In our study, the observed changes in GM asymmetry have primarily been associated with motor dysfunction, emphasizing the importance of monitoring GM asymmetry as a reflection of motor control mechanisms. Notably, the correlation analysis in the putamen has revealed that its changes are related not only to motor dysfunction but also to cognitive decline and disease progression. This finding aligns with research in Parkinson's disease [47], where it has been suggested that, with disease progression, ReHo activity in the left dorsal rostral putamen decreases, potentially leading to associative learning impairments. These findings imply that GM asymmetry, particularly in the putamen, may serve as potential biomarkers for assessing disease progression in SCA3 patients. The strength of these correlations suggests that monitoring these biomarkers could enhance our ability to predict functional decline and tailor therapeutic interventions accordingly.

The pathogenesis of neurodegenerative diseases may also present asymmetrically, with the asymmetric spread and aggregation of abnormal proteins being one possible explanation. In neurodegenerative diseases such as PD, AD, and ALS, aggregates of abnormal proteins, including α‐synuclein (α‐syn), β‐amyloid (Aβ), and TDP‐43, exhibit a markedly asymmetric distribution patterns in the brain [48, 49, 50, 51, 52, 53, 54]. This distribution is closely related to the clinical manifestations of these diseases. Additionally, in SCA3, the abnormal accumulation of ATXN3 protein may result in analogous asymmetric aggregation. Structural and functional abnormalities of ATXN3 protein result in its uneven deposition in neurons, potentially leading to cellular dysfunction and cell death [54, 55]. This asymmetry may be associated with symptoms of dyskinesia in SCA3, although the precise mechanisms remain unclear. Another potential explanation could be the asymmetry in neurotransmitter distribution. Hemispheric differences in neurotransmitter receptor and transporter density are generally positively associated with hemispheric differences in cortical thinning during the development [56]. Moreover, in dementia with Lewy bodies [57], interhemispheric asymmetry of cholinergic terminals has been linked to asymmetric brain metabolism and lateralized motor function. These mechanisms suggest that changes in GM volume involve multiple pathways. Future research should further explore the molecular mechanisms underlying the observed GM volume asymmetry and investigate how this asymmetry interacts with disease progression and clinical symptoms.

This study has several limitations. First, the present study is a cross‐sectional clinical study, which limits our ability to infer causality between disease progression and asymmetry changes. To address this, future studies should incorporate larger sample sizes and adopt a longitudinal approach to monitor changes in cerebral asymmetry over time and assess its effects on motor and nonmotor functions. This would provide a stronger basis for confirming the robustness of GM asymmetry as a potential clinical biomarker. Additionally, although this study focused on structural changes, future research should incorporate diffusion tensor imaging (DTI) and functional magnetic resonance imaging (fMRI) to explore affected neural pathways and networks, enhancing our understanding of structural asymmetry and its functional implications. Furthermore, this study did not include specific quantitative assessments of symptom asymmetry, such as the nine‐hole peg test and digital finger‐tapping test. Employing more systematic and advanced clinical tools in future research could facilitate the evaluation of the correlation between GM asymmetry and symptom asymmetry, potentially uncovering more subtle motor and functional imbalances in SCA3 patients. Lastly, the molecular mechanisms driving brain asymmetry were not investigated. Future studies should examine genetic, epigenetic, and molecular factors to uncover the underlying causes and shed light on the neurodegenerative processes in SCA3.

## 5 Conclusions

This study aims to elucidate the distinct patterns of asymmetrical changes caused by cerebellar atrophy and explore their interaction with disease progression and motor dysfunction in SCA3 patients. The findings reveal that these asymmetrical changes are not confined to the cerebellum but also extend to the visual cortex and broader neural circuits, such as the CSTC circuit. These findings offer important insights for treatment planning, suggesting that the intensity of interventions could be tailored according to the degree of asymmetry observed in each patient. Future research should explore the asymmetrical changes in brain networks and white matter fiber connections, as well as investigate the underlying molecular mechanisms, including the causes of these asymmetries and their impact on disease progression.

## Author Contributions

Chen Liu, Linfeng Shi, Jian Wang, Peiling Ou, and Lan Ou designed the study, analyzed the data, and wrote the main manuscript; Yonghua Huang and LiMeng Dai collected the data; Xingang Wang and Lihua Deng organized the data; Li Gui and Bijia Wang conducted the scale evaluation; and Guolin Ma revised the manuscript. All authors contributed to the article and approved the submitted version.

## Disclosure

The authors have nothing to report.

## Consent

All participants provided written informed consent to participate in the study.

## Conflicts of Interest

The authors declare no conflicts of interest.

## Supporting information


Data S1.

## Data Availability

The data that support the findings of this study are available from the corresponding author upon reasonable request.
